# Obsessive-Compulsive Symptoms, Polygenic Risk Score, and Thalamic Development in Children From the Brazilian High-Risk Cohort for Mental Conditions (BHRCS)

**DOI:** 10.3389/fpsyt.2021.673595

**Published:** 2021-06-07

**Authors:** Ana Beatriz Ravagnani Salto, Marcos L. Santoro, Marcelo Q. Hoexter, Andrea Parolin Jackowski, Pedro M. Pan, Maria Conceição Rosário, Sintia I. Belangero, Pedro Gomes Alvarenga, Victoria Fogaça Doretto, Afonso Mazine Tiago Fumo, Marcelo C. Batistuzzo, Pedro Macul Ferreira de Barros, Kiara R. Timpano, Vanessa K. Ota, Luis Augusto Rohde, Euripedes Constantino Miguel, James F. Leckman, André Zugman

**Affiliations:** ^1^Department and Institute of Psychiatry, Faculdade de Medicina, Universidade de São Paulo, São Paulo, Brazil; ^2^Departamento de Morfologia e Genética, Universidade Federal de São Paulo, São Paulo, Brazil; ^3^Laboratory of Integrative Neuroscience, Departamento de Psiquiatria, Universidade Federal de São Paulo, São Paulo, Brazil; ^4^Department of Methods and Techniques in Psychology, Pontifical Catholic University, São Paulo, Brazil; ^5^Department of Psychology, University of Miami, Coral Gables, FL, United States; ^6^Attention-Deficit/Hyperactivity Disorder and Developmental Psychiatry Programs, Hospital de Clínicas de Porto Alegre, Universidade Federal Do Rio Grande Do Sul, Porto Alegre, Brazil; ^7^Yale Child Study Centre, Yale School of Medicine, Yale University, New Haven, CT, United States

**Keywords:** obsessive-compulsive disorder, MRI, thalamus, obsessive-compulsive symptoms, polygenic risk score, OCD-PRS, neuroimaging

## Abstract

**Background:** Thalamic volume measures have been linked to obsessive-compulsive disorder (OCD) in children and adolescents. However, it is unclear if alterations in thalamic volumes occur before or after symptom onset and if there is a relation to the presence of sub-clinical obsessive-compulsive symptoms (OCS). Here, we explore the relationship between OCS and the rate of thalamic volume change in a cohort of children and youth at high risk to develop a mental disorder. A secondary aim was to determine if there is a relationship between OCS and the individual's OCD polygenic risk score (OCD-PRS) and between the rate of thalamic volume change and the OCD-PRS.

**Methods:** The sample included 378 children enrolled in the longitudinal Brazilian High-Risk Cohort for Mental Conditions. Participants were assessed for OCS and the symmetrized percent change (SPC) of thalamic volume across two time-points separated by 3 years, along with the OCD-PRS. Zero-altered negative binomial models were used to analyze the relationship between OCS and thalamic SPC. Multiple linear regressions were used to examine the relationship between thalamic SPC and OCD-PRS.

**Results:** A significant relationship between OCS and the right thalamus SPC (*p* = 0.042) was found. There was no significant relationship between changes in thalamic volume SPC and OCD-PRS.

**Conclusions:** The findings suggest that changes in the right thalamic volume over the course of 3 years in children may be associated to OCS. Future studies are needed to confirm these results and further characterize the specific nature of OCS symptoms associated with thalamic volumes.

## Introduction

Obsessive-compulsive disorder (OCD) is a common, chronic and potentially disabling condition (1). Obsessive-compulsive symptoms (OCS) are linked to OCD both by epidemiological and genetic studies, and have been associated with distress and impairment at the subclinical level (2, 3). Subclinical OCS in childhood increase the risk of full-blown OCD in adulthood (4). A recent study found a lifetime prevalence of OCS in children and adolescents from 11 to 21 years-old of 38.2% and the prevalence of OCD of 3% (5). The OCS prevalence varies from 8.7 to 38.2%, depending on the population and methodology used (2, 4–7). Only a minority of individuals with OCS in the community fulfill diagnostic criteria for OCD, and, indeed, the prevalence of OCD is much lower (2–3%) than OCS when both are assessed in the same sample (4, 5).

Cortico-striato-thalamo-cortical circuitry (CSTC) has been consistently implicated in the pathobiology of OCD both from animal models and neuroimaging studies (8, 9). It is hypothesized that alterations in CTSC circuits involved in sensorimotor, cognitive, affective, and motivational processes contribute to the pathophysiology of OCD (8, 10). It is likely that the same circuit is involved in OCS for individuals with subclinical OCD (11, 12). The thalamus, as part of this circuitry, is a region that has been repeatedly examined in both human and animal studies. There have been consistent findings of structural alterations in the thalamus of OCD patients in both adults and children. While four earlier meta-analyses and one mega-analysis showed no difference in thalamic volume between OCD patients and healthy controls (HC) (13–17), these studies combined adults and children and more recent work suggests that there may in fact be an association. A worldwide mega and meta-analysis from the ENIGMA-OCD Working Group reported increased thalamic volumes in unmedicated children with OCD, but no differences were seen in adult patients (18). A recent study found increased thalamic volumes in children from the community with probable OCD (19). An additional meta-analysis combining both children and adults reported that increased thalamic volumes were associated with OCD (20).

Given this literature, the thalamic volume has emerged as a potential candidate for an endophenotype for OCD and potentially OCS; however, this still needs further investigation. An endophenotype is a biological or psychological trait that is in the causal chain between genetic susceptibility and disease expression. Specific criteria have been proposed to define endophenotypes, such that the trait needs to be: associated with illness, heritable, primarily state independent, co-segregate within families, and found in unaffected family members at a higher rate than in the general population (21). Although the thalamus has been implicated in the neurobiology of OCD, it is still unknown if the thalamic alterations are related to the genetic risk for OCD, if they precede or develop after symptom's expression or if they develop after the full syndrome is present. Prospective cohorts are ideal to clarify these issues and add to the understanding of thalamic volume as an endophenotype for OCS. There is evidence that during brain development, the thalamus follows a curvilinear trajectory of volume change (22). The trajectory peaks at 13.8 years in females and at 17.4 years in males (23). Previous studies assessing thalamic volume on OCD or OCS have been limited to cross-sectional samples. To date, there is little evidence if the alteration in thalamic volumes seen in OCD and OCS are due to different trajectories that occur during childhood and adolescence or due to higher baseline volumes. It is possible that children at risk for OCD have an altered thalamic trajectory leading to the increased thalamic volume reported in unmedicated children with OCD (18). As most studies, such as the ones included in the ENIGMA mega and meta-analysis (18), are cross-sectional, the higher volumes could be related with steeper slopes leading to higher thalamic volume.

It is widely known that genetics play an important role in mental disorders. Although heritability for OCD is not very high (~0.47), it still indicates that genetic factors contribute to the etiology of this disorder (24). The heritability of OCS is reported to be 0.40, indicating that it also has a genetic basis (25, 26). Polygenic risk scores (PRS) have emerged as a potentially valuable tool for assessing genetic risk and can be useful for testing the relationship between genetic risk and endophenotypes. For common polygenic conditions like Alzheimer disease, coronary artery disease and type 2 diabetes mellitus, the PRS are being studied as a useful tool for prioritization of preventive interventions and screening, prediction of age of disease onset, benefit from lifestyle modifications and changes in clinical decision-making (27). PRS are computed from genome wide association studies (GWAS). PRS is a weighted sum of the number of risk alleles carried by an individual, in which the risk alleles and their weights are defined by the loci and their measured effects as found by GWAS (27). Genetic overlap between OCD and OCS is suggested by the fact that PRS based on OCD GWAS data significantly predicted OCS (3, 26).

A prospective longitudinal study enriched for children at risk for developing OCD is an ideal approach for studying the development, genetic risk, and neurobiology of the disorder. There are many studies comparing the neurobiology of OCD and healthy controls, but prospective longitudinal studies on the development of OCD are rare. The Brazilian High-Risk Cohort for Mental Conditions is an ongoing study that has prospective data on genetics, neuroimaging, and psychopathology. Data from the cohort was used here to study the OCS phenotype prospectively in a community-based sample.

We hypothesized that youth with subclinical OCS from a community sample and youth with genetic risk for developing OCD would have an altered rate of thalamic volume change, with slower decrease or faster increase of thalamic volume. More specifically, we hypothesized that thalamic volume symmetrized percent change (SPC) would be related to the presence and intensity of OCS in children in the follow-up. A second hypothesis was that OCS would be related to OCD-PRS. A third hypothesis was that the thalamic volume SPC would be related to OCD-PRS.

## Methods

### Participants

A subsample of the participants from the Brazilian High-Risk Cohort for Mental Conditions was included in this analysis (28). The study was submitted and approved by the Ethical Committee of the University of São Paulo. All parents signed informed consent and children provided verbal assent. Details about the cohort can be found elsewhere (28). In 2010, children from 6 to 14 years old from 57 schools in the cities of São Paulo and Porto Alegre were enrolled in the study based on their risk for mental disorders. A subsample of 750 children completed a scanning protocol. After quality control assessment, the final subsample for the current report consisted of 732 children. The baseline evaluation included a structured household interview with a biological parent, acquisition of T1-weighted brain magnetic resonance imaging (MRI) in two centers using a 1.5T General Electric Scanner and blood samples collection. After 3 years, parents were once again interviewed, and children were interviewed for psychiatric symptoms by certified psychologists. After the psychopathological assessment, participants were invited to complete another scanning session. Retention at follow-up was 90% for the psychopathological measures. In total, 378 children were included in the analysis in the present study as they were scanned in both waves of the data collection and had viable data for analysis. The primary reason some children could not undergo scanning at the second time point was due to fMRI contraindications (e.g., wearing braces), with issues such as refusal or loss to follow-up only explaining a minority of the missing wave 2 imaging data.

### Obsessive-Compulsive Symptoms

Psychiatric diagnosis was assessed using the Brazilian Portuguese version (29) of the Development and Well-Being Assessment (DAWBA) (30). This structured interview was administered to biological parents by trained lay interviewers on baseline and follow-up and to children by certified psychologists on follow-up. The interview information was then scored by trained psychiatrists who were supervised by a senior child psychiatrist to generate DSM-IV diagnosis. There were only a few participants of the cohort that developed sufficient OCS to meet the DSM-IV criteria for OCD. From the 378 children, only 1 child was diagnosed with OCD at baseline and 5 at follow-up. A dimensional score for OCS was computed using answers from the youth at follow-up using the 9 items from section F of the DAWBA (Supplementary Material). Each item is scored from 0 (no), 1 (a little) to 2 (a lot). A total score was calculated by summing the 9 items, resulting in a total OCS score from 0 (no symptoms) to 18 (maximum symptoms).

OCS score was built based on the DAWBA assessment of youth at follow-up because that was the only measurement of self-reported symptoms. The OCS score was used as the dependent variable in the hurdle models. A score for baseline OCS was computed to be used as a control variable. The baseline OCS score was computed using the same approach as the OCS score but only included information from the guardian.

An additional analysis was performed using the obsessive-compulsive (OC) factor score recently published by our group (31). Briefly, OC factor score combining information from parents and children on the follow-up was built under the Bartlett's method. The OC factor score included information from three sources: the DAWBA assessment of youth, DAWBA assessment with information from parents and Child Behavior Checklist (CBCL) information from parents. In that study, a baseline OC factor score was computed based on information only from parents combining DAWBA and CBCL. These previously reported OC factor scores were used in the mixed effects model considering that they may represent the same latent variable.

### Neuroimaging

Identical imaging protocols were used in both sites with 1.5-T MRI scanners (GE Signa HDX and GE Signa HD; GE, USA). At follow-up, children were rescanned in the same scanner as baseline at each site. T1-weighted scans (three-dimensional fast spoiled gradient sequence) used the following parameters: 160 axial slices for whole brain coverage, TR = 10.9 ms, TE = 4.2 ms, thickness = 1.2 mm, flip angle = 15°; matrix size = 256, FOV = 24 cm, and NEX = 1. Imaging acquisitions were repeated whenever participants moved during the procedure in order to ensure that optimal quality was obtained.

The T1-weighted scans were processed using FreeSurfer version 6.0. The longitudinal processing stream of Freesurfer was used to reduce variability and avoid over-regularization (32). The thalamus was selected as the region of interest and thalamic volume was computed using the automated subcortical segmentation stream of Freesurfer (33). For assessing the longitudinal change in thalamic volume, the Symmetrized Percent Change (SPC) rate computed in FreeSurfer was used. The SPC is computed using the formula: SPC = 100 ^*^ *rate*/*average*. *Rate* corresponds to the difference in volume per time unit, so rate = (volume 2 – volume 1)/(time 2 – time 1). *Average* corresponds to the average volume: average = 0.5 ^*^ (volume 1 + volume 2). The SPC could be negative, zero, or positive.

### Polygenic Risk Score

From the subsample of 378 children who had both time-points of brain imaging, 364 were genotyped. Genomic DNA was isolated from saliva (Oragene) using prepIT-L2P reagent (DNAgenotek). Genotyping was performed using the Global Screening Array (Illumina). Single-nucleotide polymorphisms (SNPs) with a minor allele frequency <1%, locus missingness >10%, or Hardy-Weinberg equilibrium significance <0.000001 were excluded, as were individuals with genotype missingness >10% and an estimation of identity by descent >0.12.

OCD polygenic risk scores (OCD-PRS) were calculated with the PRSice V2 software package (34), using as a training sample the summary statistics of the meta-analysis from the two OCD consortia, totalizing 2,688 cases (35–37). For the main analyses, *p*-threshold of 0.476 was selected, which contained 97,413 independent SNPs in the training and target samples. This *p*-threshold was pointed by PRSice V2 as the most correlated with OCS in our sample.

### Statistical Analysis

**Figure 1:**
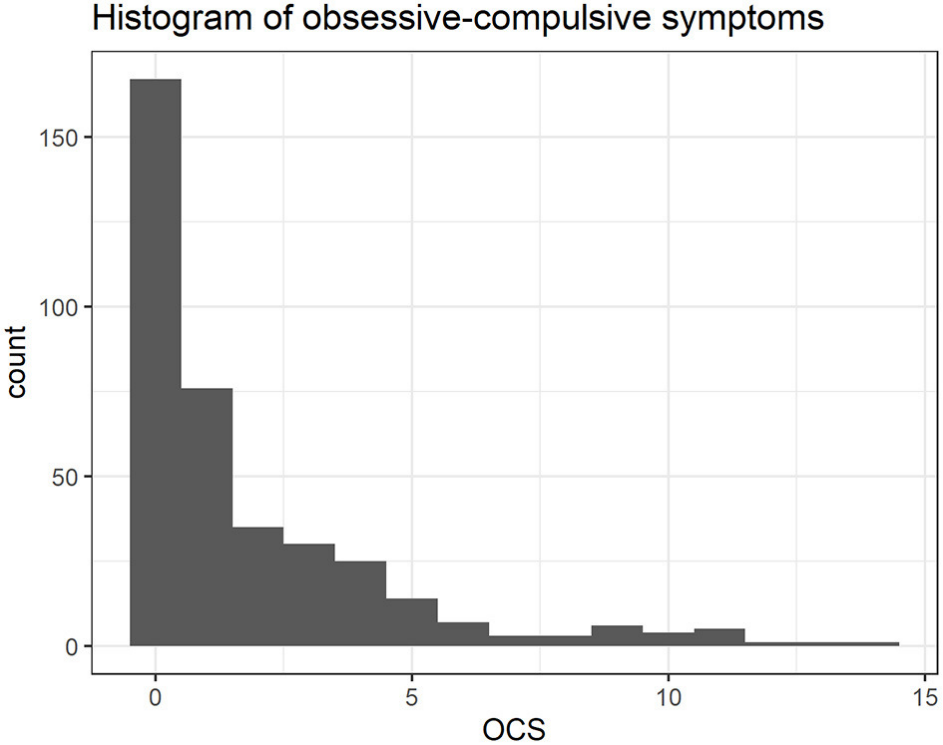
Distribution of obsessive-compulsive symptoms (OCS) at follow-up.

The variable OCS was zero inflated, with ~44% of the sample endorsing no symptoms of OCD in the DAWBA at follow-up (Figure 1). Given that, and considering that the OCS variable was skewed and over dispersed (the variance is almost 4 times the mean), we selected a model to deal with zero inflated data. A zero altered negative binomial model (ZANB)—also known as hurdle model or two-part model—was used (38). The Negative Binomial model was selected over Poisson because the latter assumes the variance is equal to the mean with equi-dispersion. After running the two models and comparing them with the likelihood ratio test of nested models (implemented in “*r*” package “lmtest”), there was significant evidence for a better fit of the Negative Binomial model compared to Poisson regression model (df = 1, χ^2^ = 137.2, *p* < 0.0001). The hurdle model considers that the entire group of participants is at risk for the event under study and that all zeros are generated from a single process (39). Hurdle models consist of two parts. In the first part (zero-hurdle), the data are considered as zeros vs. non-zeros and a binomial model is used to model the probability that a zero value is observed (40). In the second part (count), the non-zero observations are modeled with a truncated negative binomial model. Two ZANB models were built: one for the left and one for the right thalamus. The models included the OCS as dependent variable, thalamic SPC as the independent variable and age at follow-up, sex, any psychiatric comorbidity (excluding OCD), and site as covariates. The same variables were included in the zero-hurdle and count components of the models. To control for baseline OCS, a model was built with the same variables listed above, but including OCS at baseline as an independent variable (Supplementary Table 1). Another ZANB model was built to access the relationship between OCS and OCD-PRS, including age at follow-up, sex, any psychiatric comorbidity (excluding OCD), site and 10 first principal components from genetic data as covariates.

For the analysis of the relationship between thalamic volume SPC and OCD-PRS multiple linear regression was used. The regressions included the thalamic SPC as the dependent variable, OCD-PRS as the independent variable and age at follow-up, sex, site, and 10 first principal components from genetic data as covariates.

A mixed effects model was used to assess total thalamic volume change in relation to OC factor scores reported previously (31). The mixed model was run with the participant entered as random factor and thalamic volume as dependent variable. The OC factor score was the independent variable and included the assessment at baseline and follow-up as described in the “obsessive-compulsive symptoms” section. Age, an interaction term between OC factor score and age, sex, any psychiatric comorbidity (excluding OCD), total intracranial volume and site were included as covariates.

To study the relationship between thalamic volume change and OCD-PRS, a mixed effects model was built with participant entered as random factor, thalamic volume as dependent variable and OCD-PRS as independent variable. Age, sex, site, total intracranial volume and 10 first principal components from genetic data were included as covariates.

The statistical analysis was performed using R. Version 4.0.1. The function “hurdle” from the R package “pscl” was used to build the ZANB models. The function “lm” was used for the linear model. The function “lmer” from the R package “lme4” was used to build the mixed effects models.

## Results

### Sample Characteristics

The sample for the neuroimaging evaluation consisted of 378 children who underwent two scans in a 3-year interval. The mean age at baseline was 10.48, at follow-up it was 14.24 and 42% were females (Table 1). From the 378 children, 364 had viable blood samples and took part in the genetics evaluation. The mean age for the 364 children was 10.49 at baseline, 14.25 at follow-up and 42% were females. The distribution of OCS was right-skewed and zero-inflated (Figure 1). There was around 44% of zeros.

**Table 1:** Demographic and clinical characteristics.

	**Frequency (percentage) or mean (SD) or median (IQR)**
Gender female *n* (%)	158 (42)
IQ mean (SD)	103.93 (17.26)
Age baseline mean (SD)	10.48 (1.83)
Age follow-up mean (SD)	14.24 (1.84)
Socioeconomic status baseline mean (SD)	21.59 (4.38)
Socioeconomic status follow-up mean (SD)	21.55 (4.38)
Any psychiatric disorder baseline^a^ *n* (%)	114 (30)
Any psychiatric disorder follow-up^a^ *n* (%)	106 (28)
OCD baseline^a^ *n* (%)	1 (0.3)
OCD follow-up^a^ *n* (%)	5 (1.3)
OCS median (IQR)	1 (0–3)

a *Based on the Development and Well-Being Assessment (DAWBA) questionnaire*.

*SD, standard deviation; IQR, interquartile range; IQ, intelligence coefficient; OCD, Obsessive-compulsive disorder*.

### Thalamic Volume SPC and OCS

**Figure 2:**
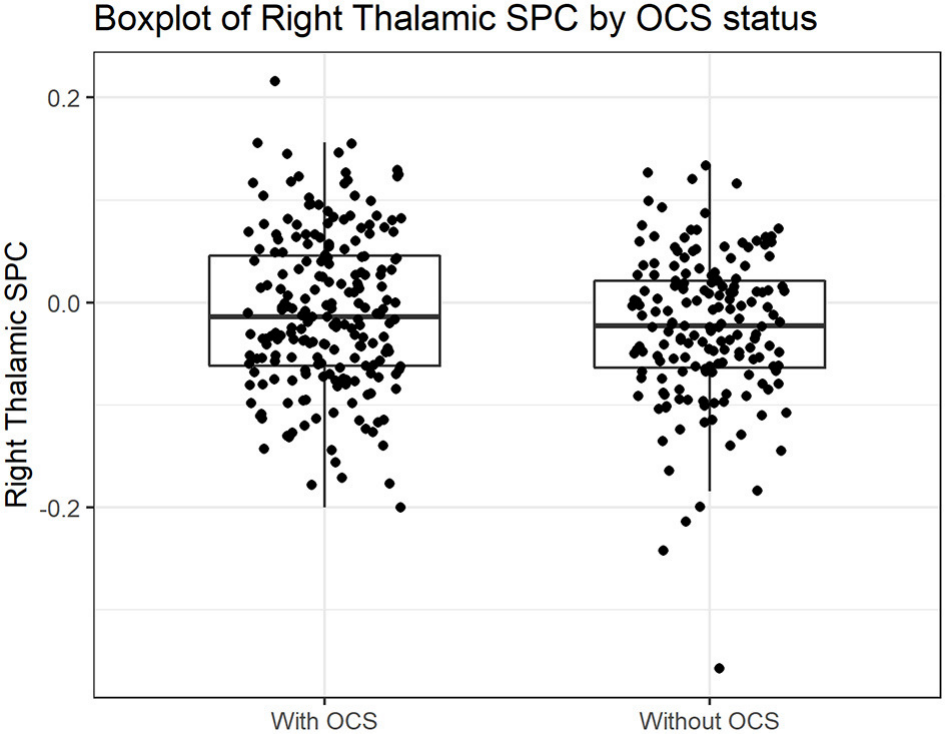
Boxplot of Right Thalamic SPC in participants with and without obsessive-compulsive symptoms at follow-up. SPC, Symmetrized Percent Change.

A significant positive relationship between right SPC and OCS (*p* = 0.042) was found in the zero-hurdle part of the model (Table 2). There was no significant relationship between right SPC and OCS in the count part of the model (Table 2). The zero-hurdle part of the model gives the probability of a non-zero count (40). The results indicate that right thalamus change was related to having at least one OCS reported (Figure 2). The thalamic variation was not related to the amount of OCS as the count part was not significant. There was no significant relationship between left SPC and OCS in both the zero-hurdle and the count parts of the model (Table 2). The analysis was repeated including OCS reported by parents at baseline as a control variable and the relationship between right SPC and OCS remained significant (Supplementary Table 1). Models including an interaction term between both Thalamic SPC and age were tested. In those models there was no significant association between the variables (Supplementary Table 2).

**Table 2:** Zero-altered negative binomial (ZANB) models examining the relationship between OCS and thalamic SPC.

**Right thalamus**			**Left thalamus**		
**Count model**	***B***	***p*-value**	**Count model**	***B***	***p*-value**
Right thalamic SPC	1.020	0.469	Left thalamic SPC	−0.575	0.700
Sex	−0.064	0.744	Sex	−0.133	0.523
Age	−0.061	0.255	Age	−0.076	0.159
Site	0.275	0.147	Site	0.296	0.123
Comorbidity	0.177	0.392	Comorbidity	0.174	0.402
**Zero-hurdle model**	***B***	***p*****-value**	**Zero-hurdle model**	***B***	***p*****-value**
Right thalamic SPC	3.161	0.042^*^	Left thalamic SPC	2.791	0.085
Sex	0.161	0.461	Sex	0.174	0.434
Age	0.021	0.728	Age	0.021	0.726
Site	0.394	0.064	Site	0.356	0.098
Comorbidity	0.314	0.195	Comorbidity	0.300	0.213

*SPC, Symmetrized Percent Change; OCS, obsessive-compulsive symptoms*.

* *p < 0.05*.

### OCD-PRS and OCS

There was no significant relationship between OCD-PRS and OCS in both the zero-hurdle and the count parts of the model (Supplementary Table 3).

### Thalamic Volume SPC and OCD-PRS

There was no significant relationship between right or left thalamic SPC and OCD-PRS (Supplementary Table 4).

### Mixed Effects Model for Total Thalamic Volume

There was no significant relationship between thalamic volume and OC factor scores, assessed longitudinally (Supplementary Table 5).

There was no significant relationship between thalamic volume and OCD-PRS in the mixed model (Supplementary Table 6).

## Discussion

Changes in right thalamic volume after 3 years were related to the presence of OCS at follow-up in children from a community sample. This result was only significant in the zero-hurdle part of the model. No relationship was observed between changes in left thalamic volume and OCS or between both right and left thalamic volume change and OCD-PRS. The Thalamic SPC measures change, ranging from negative to positive values (Figure 2). A thalamic volume decrease (thalamic volume in the follow-up is smaller than baseline), will yield a negative SPC. On the other hand, participants with an increase in thalamic volume in the period will have a positive SPC. Our results indicate that participants with at least one OCS had a slower decrease in right thalamic volume.

Previous studies, as the recent worldwide mega and meta-analysis from the ENIGMA-OCD Working Group and one meta-analysis, found increased thalamic volume in individuals with OCD (18, 20). It is possible that a slower decrease in right thalamic volume found in our sample could lead to increased average thalamic volume found in previous studies. It can be hypothesized that individuals from the community with subclinical OCS might have a slower decrease in thalamic volume suggesting that thalamic trajectory alterations might be found even without the full syndrome. However, five meta-analyses found no differences in thalamic volume comparing individuals with OCD and HC (13–17). Our analysis of thalamic volume was limited by the fact that self-reported OCS was only assessed on follow-up. The analysis including other informants by using the OC factor score did not support an altered thalamic volume related to symptoms in our sample. Of note, there are important differences in the samples from the prior studies included in previous meta-analyses and the sample of participants included in this study. The sample in the present study is community based and the prevalence of OCD was very low. Moreover, it is not known at this time how many participants will develop full-blown OCD later in young adulthood. Despite not fulfilling the diagnostic criteria and having relatively low levels of symptom severity, subclinical OCS have been associated with psychiatric comorbidities and impaired functioning in the literature (5, 41, 42). A recent neuropsychological study of children and adolescents with OCS that were first-degree relatives of an individual with OCD identified impairments in spatial working memory and a trend in significance for impairment in motor and processing speed (43). Here we found that the presence of OCS may be directly associated with right thalamic volumes change, and the thalamus is a key element of the cortico-striato-thalamo-cortical (CSTC) circuits that are implicated in the pathophysiology of OCD (44). The thalamus selectively filters information for further processing in other areas of the brain, functioning as a relay station and a gatekeeper (45). It has a role in the processing of information related to many cognitive processes, including motor processes, cognition, emotion, learning, pain, attention, and consciousness (45).

The polygenic risk score (PRS) has been related to the genetic risk for developing OCD. We expected to find an association between OCS and OCD-PRS and between thalamic volume change and OCD-PRS, but no relationship was detected. One hypothesis for that finding is the small sample size of OCD GWAS studies. Therefore, future summary statistics may be more informative. GWAS for chronic clinical conditions like coronary artery disease and type 2 diabetes include >100,000 of individuals (46, 47). Even for some psychiatric disorders like Alzheimer's disease and major depressive disorder there are around 100,000 individuals in the GWAS (48, 49). For OCD, there are only two GWAS studies published totalizing 2,688 cases (35–37). The small sample size of GWAS for OCD underpower the ability of OCD-PRS to explain the OCD phenotype. Moreover, OCD GWAS are based mostly on European ancestry samples and our sample is ethnically diverse as the Brazilian population has a multi-ethnic and admixed background. It has been previously shown that the performance of PRS in non-European populations is generally poorer than the performance in European ancestry samples, particularly for African ancestry samples (50). Despite considering ancestry by using 10 principal components, we must consider that the training and target samples used to build PRS are from different populations, and, hence, this could still have had an important impact (51).

Results should be interpreted in light of several limitations. Only participants with complete assessment in baseline and follow-up were included, as we were interested in the rate of change of thalamic volume. This decreased the available sample for this study. Another limitation was the small number of participants that met DSM-IV criteria for OCD in the sample. Differently from previous OCD studies, the phenotype here is based only on the presence of OCS. Small changes in symptom dimensions are likely to be related to small effect sizes, hence, there is a clear need to increase the sample size to achieve adequate statistical power.

This is the first study that assessed longitudinal changes during thalamic development in youth with OCS. In this study, thalamic alterations reported in OCD patients were found in youth with OCS. It can be hypothesized that thalamic alterations may be a trait related to the OCS phenotype and independent of disease state, supporting the idea of an endophenotype. However, the hypothesis that individuals with genetic risk for OCD would also show thalamic alterations was not confirmed. Thus, thalamic alterations may not be related to increased genetic risk for OCD. However, alterations in thalamic development may be a marker of OCS even before the full syndrome develops.

## Data Availability Statement

The raw data supporting the conclusions of this article will be made available by the authors, without undue reservation.

## Ethics Statement

The studies involving human participants were reviewed and approved by Ethical Committee of the University of São Paulo. Written informed consent to participate in this study was provided by the participants' legal guardian/next of kin.

## Author Contributions

AR, AZ, MS, MH, EM, JL, PP, MR, PA, PM, and MB conceived and designed the analysis. AZ, AJ, JL, EM, and MH supervised the research. MS, MH, AJ, PP, SB, and VO collected the data. AR, MS, VO, and AZ performed the analysis. AR, AZ, MS, and JL wrote the manuscript. LR, VD, PM, PA, MB, SB, VO, AF, and KT provided revisions to scientific content of the manuscript. All authors discussed the results and reviewed the final manuscript.

## Conflict of Interest

The authors declare that the research was conducted in the absence of any commercial or financial relationships that could be construed as a potential conflict of interest.
