# Supplementary material for: Obsessive-Compulsive Symptoms, Polygenic Risk Score, and Thalamic Development in Children From the Brazilian High-Risk Cohort for Mental Conditions (BHRCS)
Source: Front Psychiatry. 2021 Jun 7;12:673595. doi: 10.3389/fpsyt.2021.673595 (PMC8215160; doi:10.3389/fpsyt.2021.673595)
Supplement: Supplementary file 1 [file Data_Sheet_1.docx]

Supplementary Material

# Obsessive-compulsive symptoms (OCS) score

The OCS score was computed based on the sum of the following items from the section F of the DAWBA:

F2: Over the last 4 weeks have you had any of the following rituals (doing any of the following things over and over again even, though you have already done them or don’t need to do them at all)?

a) Excessive cleaning: hand washing, baths, showers, toothbrushing etc.?

b) Other special measures to avoid dirt, germs or poisons?

c) Excessive checking: electric switches, gas taps, locks, doors, the oven?

d) Repeating the same simple activity many times in a row for no reason, e.g. repeatedly standing up or sitting down or going backwards and forwards through a doorway?

e) Touching things or people in particular ways?

f) Arranging things so they are just so, or exactly symmetrical?

g) Counting to particular lucky numbers or avoiding unlucky numbers?

F3: Over the last 4 weeks have you been obsessively worrying about dirt, germs or poisons – not being able to get thoughts about them out of your mind?

F4: Over the last 4 weeks have you been obsessed by the worry that something terrible will happen to yourself or to others - illnesses, accidents, fires etc.

The 9 items vary from score 0 (no), 1 (a little) to 2 (a lot). For the computed OCS the minimum score is 0 and the maximum is 18.

# Supplementary Tables

**Supplementary Table 1.** Zero-altered negative binomial (ZANB) models examining the relationship between OCS and Thalamic SPC with baseline OCS reported by parents

| **Right Thalamus** | | | **Left Thalamus** | | |
| --- | --- | --- | --- | --- | --- |
| **Count model** | **B** | **p-value** | **Count model** | **B** | **p-value** |
| Right Thalamic SPC | 1.120 | 0.429 | Left Thalamic SPC | -0.468 | 0.757 |
| Sex | -0.056 | 0.775 | Sex | -0.123 | 0.555 |
| Age | -0.056 | 0.292 | Age | -0.073 | 0.183 |
| Site | 0.255 | 0.185 | Site | 0.279 | 0.153 |
| Comorbidity | 0.179 | 0.385 | Comorbidity | 0.176 | 0.396 |
| OCS baseline | -0.048 | 0.554 | OCS baseline | -0.036 | 0.661 |
| **Zero-hurdle model** | **B** | **p-value** | **Zero-hurdle model** | **B** | **p-value** |
| Right Thalamic SPC | 3.068 | 0.049* | Left Thalamic SPC | 2.662 | 0.101 |
| Sex | 0.155 | 0.481 | Sex | 0.165 | 0.458 |
| Age | 0.019 | 0.758 | Age | 0.019 | 0.759 |
| Site | 0.433 | 0.045* | Site | 0.397 | 0.070 |
| Comorbidity | 0.304 | 0.209 | Comorbidity | 0.291 | 0.228 |
| OCS baseline | 0.100 | 0.291 | OCS baseline | 0.099 | 0.296 |

SPC = Symmetrized Percent Change, OCS = obsessive-compulsive symptoms

*p<0.05

**Supplementary Table 2.** Zero-altered negative binomial (ZANB) models examining the relationship between OCS and Thalamic SPC with interaction term between Thalamic SPC and age

| **Right Thalamus** | | | **Left Thalamus** | | |
| --- | --- | --- | --- | --- | --- |
| **Count model** | **B** | **p-value** | **Count model** | **B** | **p-value** |
| Right Thalamic SPC | -9.123 | 0.395 | Left Thalamic SPC | -11.456 | 0.287 |
| Sex | -0.016 | 0.938 | Sex | -0.099 | 0.636 |
| Age | -0.054 | 0.312 | Age | -0.067 | 0.217 |
| Site | 0.246 | 0.199 | Site | 0.263 | 0.175 |
| Comorbidity | 0.178 | 0.387 | Comorbidity | 0.172 | 0.403 |
| Right Thalamic SPC*Age | 0.719 | 0.341 | Left Thalamic SPC*Age | 0.782 | 0.308 |
| **Zero-hurdle model** | **B** | **p-value** | **Zero-hurdle model** | **B** | **p-value** |
| Right Thalamic SPC | 6.215 | 0.623 | Left Thalamic SPC | 8.956 | 0.453 |
| Sex | 0.155 | 0.481 | Sex | 0.163 | 0.466 |
| Age | 0.017 | 0.785 | Age | 0.014 | 0.825 |
| Site | 0.397 | 0.062 | Site | 0.362 | 0.094 |
| Comorbidity | 0.314 | 0.195 | Comorbidity | 0.297 | 0.218 |
| Right Thalamic SPC*Age | -0.216 | 0.808 | Left Thalamic SPC*Age | -0.435 | 0.601 |

SPC = Symmetrized Percent Change, OCS = obsessive-compulsive symptoms

**Supplementary Table 3.** Zero-altered negative binomial (ZANB) model examining the relationship between OCS and OCD-PRS

| **Count model** | **B** | **p-value** |
| --- | --- | --- |
| OCD-PRS | -889.8 | 0.437 |
| Sex | -0.159 | 0.394 |
| Age | -0.042 | 0.402 |
| Site | 0.327 | 0.103 |
| Comorbidity | 0.334 | 0.102 |
| **Zero-hurdle model** | **B** | **p-value** |
| OCD-PRS | -1064 | 0.418 |
| Sex | -0.055 | 0.803 |
| Age | 0.007 | 0.903 |
| Site | 0.389 | 0.098 |
| Comorbidity | 0.252 | 0.322 |

Ten first principal components from genetic data included in the model

OCS = obsessive-compulsive symptoms, OCD = Obsessive-Compulsive Disorder, PRS = Polygenic Risk Score

**Supplementary Table 4.** Multiple linear regression examining the relationship between Thalamic SPC and OCD-PRS

| **Right Thalamus** | | | **Left Thalamus** | | |
| --- | --- | --- | --- | --- | --- |
|  | **B** | **p-value** |  | **B** | **p-value** |
| OCD-PRS | -17.61 | 0.691 | OCD-PRS | -21.74 | 0.610 |
| Sex | -0.029 | p<0.001* | Sex | -0.036 | p<0.001* |
| Age | -0.011 | p<0.001* | Age | -0.014 | p<0.001* |
| Site | 0.014 | 0.076 | Site | 0.030 | p<0.001* |

Ten first principal components from genetic data included in the model

SPC = Symmetrized Percent Change, OCS = obsessive-compulsive symptoms

*p<0.05

**Supplementary Table 5.** Mixed effect model examining the relationship between thalamic volume and OC factors

|  | **B** | **p-value** |
| --- | --- | --- |
| OC factors | 49.73 | 0.666 |
| Sex | -186.6 | 0.009* |
| Age | -92.87 | p<0.000* |
| Site | -46.27 | 0.479 |
| Comorbidity | -56.43 | 0.218 |
| Total intracranial volume | 0.007 | p<0.000* |
| OC factors*Age | -1.591 | 0.859 |

OC factors = obsessive-compulsive factor at baseline and follow-up

*p<0.05

**Supplementary Table 6.** Mixed effect model examining the relationship between thalamic volume and OCD-PRS

|  | **B** | **p-value** |
| --- | --- | --- |
| OCD-PRS | -115600 | 0.842 |
| Sex | -356.1 | 0.001* |
| Age | -90.15 | p<0.000* |
| Site | 77.64 | 0.452 |
| Total intracranial volume | 0.006 | p<0.000* |

Ten first principal components from genetic data included in the model

*p<0.05
